# Training Challenges for Effectively Implementing a Technology Clinical Trial: A Snapshot From PRISM 2.0

**DOI:** 10.1093/geroni/igab046.1194

**Published:** 2021-12-17

**Authors:** Wendy Rogers, Tracy Mitzner, Kara Cohen, Jerad Moxley

**Affiliations:** 1 University of Illinois Urbana-Champaign, Champaign, Illinois, United States; 2 Georgia Institute of Technology, Atlanta, Georgia, United States; 4 Weill Cornell Medicine, New York, New York, United States

## Abstract

Technology interventions can only be adequately assessed for efficacy if participants are adequately trained to use the technology. Only then can an evaluation be made about whether the technology intervention affects the outcome of interest. In the PRISM study, our goal was to teach inexperienced older adults to use either a tablet computer (control) or the PRISM 2.0 system. In this presentation we will discuss the training processes we used for both groups (e.g., segmenting sessions, providing homework, observations), to enable us to evaluate the relative benefits of PRISM for social connectedness. We will describe the training challenges and the need for assessors to be able to troubleshoot technology issues. We will evaluate individual differences in training success and drop-outs to provide insights for other technology intervention studies. Understanding these individual differences can provide guidance for the deployment of new technologies that may benefit health, social interaction, or cognitive engagement.

